# The relationship of hemoglobin concentration and signal intensity changes in oxygenation-sensitive cardiovascular magnetic resonance imaging

**DOI:** 10.1186/1532-429X-17-S1-Q110

**Published:** 2015-02-03

**Authors:** Gobinath Nadeshalingam, Dominik P Guensch, Kady Fischer, Matthias G Friedrich

**Affiliations:** 1 Philippa and Marvin Carsley CMR Research Centre, Montreal Heart Institute, Montreal, QC, Canada; 2 Anesthesiology and Pain Therapy, Bern University Hospital - Inselspital, Bern, Switzerland

## Background

Oxygenation-sensitive cardiovascular magnetic resonance (OS-CMR) has become a feasible diagnostic imaging modality for monitoring changes of myocardial oxygenation. Yet, potential confounding factors of this technique are not well understood. Due to T2 effects caused by tissue water content, the hydration status may impact signal intensity. We aimed at quantifying the confounding effect of a significant change of the hydration status in humans on the observed signal intensity (SI) in OS-CMR images.

## Methods

Eighteen healthy volunteers underwent OS-CMR using a clinical 3T MRI system. Hemoglobin (Hb) concentrations were measured at baseline and immediately following rapid crystalloid infusion of 1,000ml of Lactate Ringer's solution (LRS). OS-CMR images were acquired in a mid-ventricular short axis view. Myocardial SI was measured in end-systolic frames during a maximal voluntary breath-hold, after a 60-second period of hyperventilation. SI changes between beginning and end of breath-holds were expressed relative to baseline (% change).

## Results

**Figure 1:**
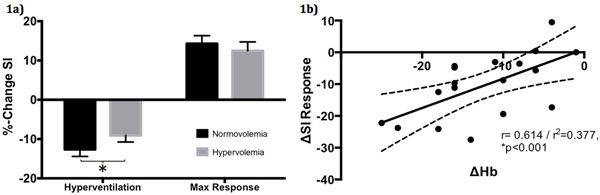
a) %-change in OS-SI at normo- and hypervolemia in response to hyperventilation and maximal apnea (*p<0.05); b) Correlation between the difference in Hb (g/L) and the difference in %SI-change between baseline and hemodilution during maximal vasodilation (r=0.61, p<0.001).

The infusion resulted in a significant decrease in measured Hb (142.0±3.6 vs. 129.7±3.6 g/L; p<0.001), while SI increased by 3.6±1.4% between baseline images at normo- and hypervolemia (p<0.05). There was a weak yet significant negative correlation between Hb concentration and SI at baseline (r=-0.48, p<0.01). For both, hyperventilation and the SI changes induced by apnea were attenuated after hemodilution (fig. 1a, p<0.05). The extent of the SI change induced by hemodilution was correlated to the change of Hb (fig. 1b).

## Conclusions

The hydration status may be a significant confounder in OS-CMR imaging. Hypervolemia leads to an increase in SI at baseline and attenuates the SI response during vasoactive breathing maneuvers. The linear correlation between Hb changes and differences in OS-SI changes may allow for establishing correction factors for Hb.

## Funding

N/A.

